# Jeyes' Disinfectants

**Published:** 1888-01-28

**Authors:** 


### JEYES' DISINFECTANTS.

THROUGH the courtesy of the Jeyes' Sanitary Compounds Company (Limited), of 43, Cannon Street, we have had forwarded for our inspection and trial a series of their preparations. These we have found very efficacious for all purposes for which such substances are in use. It is not necessary at this period to say much of disinfectants generally. Common experience has so extensively justified their use, that no well regulated establishment or household is now without them. The increasing knowledge of the community of all sanitary buestions has caused a widespread and growing demand for these articles. To meet this demand, the Jeyes Company have prepared their compounds in every variety of form. The powder for sprinkling is sold in strong tins of various sizes, which have firmly-fitting and perforated lids. When it is required to purify a dustbin, or any unpleasantness in the house or stable, the tin is a dredger ready for use, and regulates the quantity of disinfecting powder to a nicety. Similarly the liquids are in bottles of convenient size, which are as promptly available for the dressing-room, bath-room, or closet. In addition to the liquid and powder there are several varieties of soap. That for the toilet is a white soap agreeably manufactured, and without unpleasant odour. The coarser soap for the washing of floors, walls, and dirty linen is much stronger, and is a very convenient as well as a very valuable purifier. As we have said, there is no need to argue the question of the value of disinfectants; everybody is at one on that point. What is of importance to the public to know is where these can be had in every kind of form convenient for the particular use for which they are required. Jeyes' sanitary compounds are excellent in themselves, and so made up as to be immediately available for every conceivable purpose.

A WET towel wrung out and applied with vigorous friction forms a useful substitute for the sponge-bath for delicate people who shrink from the shock of immersion, and in most cases it will in time so strengthen and accustom the skin to variations of temperature as to prepare it to endure the other and more tonic bath.—*Malcolm Morris, F.R.C.S.E.*

